# *Jatropha gossypiifolia* L. (Euphorbiaceae): A Review of Traditional Uses, Phytochemistry, Pharmacology, and Toxicology of This Medicinal Plant

**DOI:** 10.1155/2014/369204

**Published:** 2014-06-05

**Authors:** Juliana Félix-Silva, Raquel Brandt Giordani, Arnóbio Antonio da Silva-Jr, Silvana Maria Zucolotto, Matheus de Freitas Fernandes-Pedrosa

**Affiliations:** ^1^Laboratório de Tecnologia & Biotecnologia Farmacêutica (TecBioFar), Programa de Pós-graduação em Ciências Farmacêuticas (PPgCF), Universidade Federal do Rio Grande do Norte (UFRN), Rua General Cordeiro de Farias, s/n, Petrópolis, 59012-570 Natal, RN, Brazil; ^2^Laboratório de Farmacognosia, Departamento de Farmácia, Universidade Federal do Rio Grande do Norte (UFRN), Rua General Cordeiro de Farias, s/n, Petrópolis, 59012-570 Natal, RN, Brazil

## Abstract

*Jatropha gossypiifolia* L. (Euphorbiaceae), widely known as “*bellyache bush,*” is a medicinal plant largely used throughout Africa and America. Several human and veterinary uses in traditional medicine are described for different parts and preparations based on this plant. However, critical reviews discussing emphatically its medicinal value are missing. This review aims to provide an up-to-date overview of the traditional uses, as well as the phytochemistry, pharmacology, and toxicity data of *J. gossypiifolia* species, in view of discussing its medicinal value and potential application in complementary and alternative medicine. Pharmacological studies have demonstrated significant action of different extracts and/or isolated compounds as antimicrobial, anti-inflammatory, antidiarrheal, antihypertensive, and anticancer agents, among others, supporting some of its popular uses. No clinical trial has been detected to date. Further studies are necessary to assay important folk uses, as well as to find new bioactive molecules with pharmacological relevance based on the popular claims. Toxicological studies associated with phytochemical analysis are important to understand the eventual toxic effects that could reduce its medicinal value. The present review provides insights for future research aiming for both ethnopharmacological validation of its popular use and its exploration as a new source of herbal drugs and/or bioactive natural products.

## 1. Introduction

The Euphorbiaceae family, which is considered one of the largest families of Angiosperms, covers about 7,800 species distributed in approximately 300 genera and 5 subfamilies worldwide. These species occur preferentially in tropical and subtropical environments [1, 2].

Among the main genera belonging to this family, there is* Jatropha* L., which belongs to the subfamily Crotonoideae, Jatropheae tribe and is represented by about 200 species. This genus is widely distributed in tropical and subtropical regions of Africa and the Americas [1]. The name “*Jatropha*” is derived from the Greek words “*jatros,*” which means “doctor” and “*trophe,*” meaning “food,” which is associated with its medicinal uses [3]. The* Jatropha* genus is divided into two subgenera,* Jatropha* and* curcas,* from which the subgenus* Jatropha* has the widest distribution, with species found in Africa, India, South America, West Indies, Central America, and the Caribbean [4].* Jatropha* species are used in traditional medicine to cure various ailments in Africa, Asia, and Latin America or as ornamental plants and energy crops [3]. Several known species from genus* Jatropha* have been reported for their medicinal uses, chemical constituents, and biological activities such as* Jatropha curcas*,* Jatropha elliptica*,* Jatropha gossypiifolia*, and* Jatropha mollissima*, among others [3].

**Figure 1:**
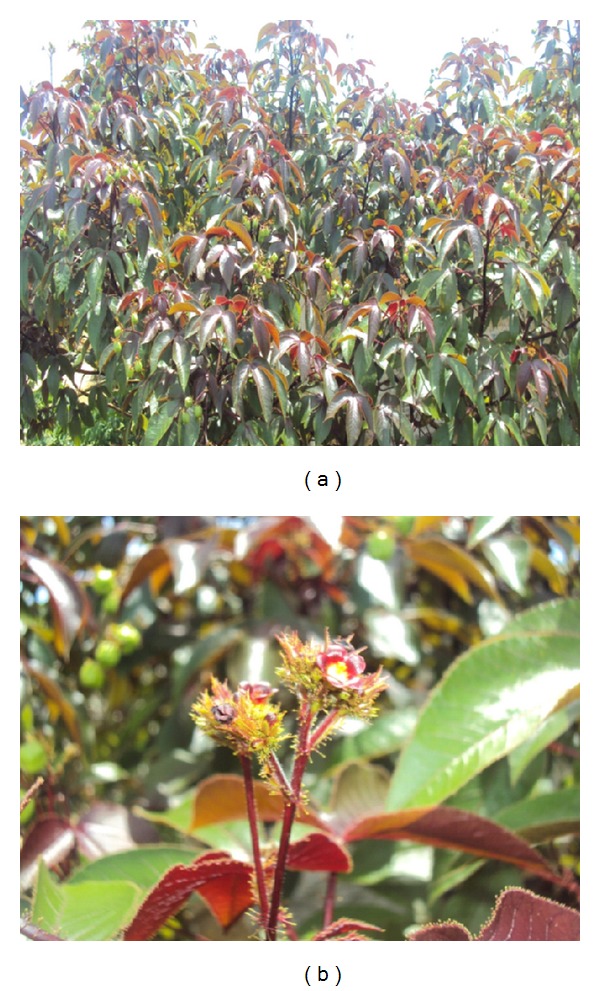
*Jatropha gossypiifolia* L. (a) aerial parts of plant. (b) flowers detail. Photography by Juliana Félix-Silva.

From these species,* Jatropha gossypiifolia* L. (Figure 1) is discussed here. It is a vegetal species widely known as “*bellyache bush*” and is a multipurpose medicinal plant largely used in folk medicine for the treatment of various diseases [3, 5, 6]. It is widely distributed in countries of tropical, subtropical, and dry tropical weather and tropical semiarid regions of Africa and the Americas [7]. In Brazil, it predominates in the Amazon, Caatinga, and Atlantic Forest and is distributed throughout the country in the North, Northeast, Midwest, South, and Southeast regions [8].

Several human and veterinary uses in traditional medicine are described for different parts (leaves, stems, roots, seeds, and latex) and preparations (infusion, decoction, and maceration, among others) based on this plant, by different routes (oral or topical). The most frequent reports concern its antihypertensive, anti-inflammatory, antiophidian, analgesic, antipyretic, antimicrobial, healing, antianemic, antidiabetic, and antihemorrhagic activities, among many other examples [3, 5, 7, 9]. Other uses are also related to this plant, such as biodiesel production, pesticide, insecticide, vermifuge, ornamentation, and even its use in religious rituals [3, 6, 10–13].

An important feature of* J. gossypiifolia* species is that, due to its important potential medicinal applications, in Brazil, it is included in the National List of Medicinal Plants of Interest to the Brazilian Public Health System (*Relação Nacional de Plantas Medicinais de Interesse ao Sistema Único de Saúde Brasileiro*—*RENISUS*), which is a report published by the Brazilian Health Ministry in February 2009 that includes 71 species of medicinal plants that have the potential to generate pharmaceutical products of interest to public health of Brazil [14].

Regarding its phytochemical constitution, alkaloids, coumarins, flavonoids, lignoids, phenols, saponins, steroids, tannins, and terpenoids were already detected in different extracts from different parts of this plant [15].

Among the main activities already studied for this species (including various types of extracts from different parts of the plant), the antihypertensive, antimicrobial, anti-inflammatory, antioxidant, and antineoplasic activities mainly stand out, supporting some of its popular uses [3, 16].

Some toxicity studies have shown that despite the known toxicity of* Jatropha* species,* J. gossypiifolia* presented low toxicity in some* in vitro* and* in vivo* experiments. However, some studies have indicated that ethanolic extract from the leaves, in acute oral use, is safe for rats, but with chronic use, it could be toxic [17–19].

So, in view of the potential applications of this plant, this review aims to provide an up-to-date overview of the traditional uses, phytochemistry, pharmacology, and toxicity data of different parts from* J. gossypiifolia*, which could be significant in providing insights for present and future research aimed at both ethnopharmacological validation of its popular use, as well as its exploration as a new source of herbal drugs and/or bioactive natural products. The medicinal value and pharmacological and/or biotechnological potential of this species are also discussed in this paper.

## 2. Methodology

An extensive review of the literature was undertaken in different national and international scientific sources, such as Centre for Reviews and Dissemination (http://www.crd.york.ac.uk/CRDWeb/), The Cochrane Library (http://www.thecochranelibrary.com), PubMed (http://www.ncbi.nlm.nih.gov/pubmed/), Science Direct (http://www.sciencedirect.com/), Scopus (http://www.scopus.com/), Lilacs (http://lilacs.bvsalud.org/), Scielo (http://www.scielo.org/php/index.php), Web of Knowledge (http://apps.webofknowledge.com), and the Brazilian database of thesis and dissertations “Domínio Público” (http://www.dominiopublico.gov.br/pesquisa/PesquisaPeriodicoForm.jsp). The study database included original articles, theses, books, and other reports that preferentially had been judged for academic quality (peer-reviewed), covering several aspects of the vegetal species (botany, phytochemistry, traditional uses, pharmacology, or toxicology), dating from 1967 (first scientific report) to November 2013, without language restriction. The search strategy was constructed based on the scientific name, synonyms, and main popular names of the species identified by the botanical databases “Flora do Brasil” (http://floradobrasil.jbrj.gov.br), Tropicos (http://www.tropicos.org), The Plant List (http://www.theplantlist.org), and NCBI Taxonomy Browser (http://www.ncbi.nlm.nih.gov/taxonomy). The search strategy contained the combination of the following terms: “*Jatropha gossypiifolia*” OR “*Jatropha gossypifolia*” OR “*Jatropha gossipyifolia*” OR “*Manihot gossypiifolia*” OR “*Adenoropium gossypiifolium*” OR “*Adenoropium elegans*” OR “*Jatropha elegans*” OR “*Jatropha staphysagriifolia*” OR* “pinhão roxo”* OR* “pinhão-roxo”* OR* “pião roxo”* OR* “pião-roxo”* OR* “peão-roxo”* OR* “peão roxo”* OR* “batata-de-teu”* OR* “bata de teu”* OR* “erva-purgante”* OR* “erva purgante”* OR* “jalapão”* OR* “mamoninha”* OR* “raiz-de-teiú”* OR* “raiz de teiú”* OR* “peão-curador”* OR* “peão curador”* OR* “peão-pajé”* OR* “peão pajé”* OR* “pião-caboclo”* OR* “pião caboclo”* OR* “black physicnut”* OR* “bellyache bush”*. The Endnote X.3.0.1 reference manager was used. The software ACD/ChemSketch Freeware Version 12.01 was used to draw the chemical structures.

## 3. Botanic Information

*Jatropha gossypiifolia* Linneus is a Euphorbiaceae plant popularly known worldwide as* “bellyache bush” *or* “black physicnut”*. It is a pantropical species originating from South America that is cultivated in tropical countries throughout the world [20–22].

In Brazil, it is known by various popular names and the most common are “*pinhão-roxo,*” “*pião-roxo,*” “*peão-roxo,*” “*batata-de-teu,*” “*erva-purgante,*” “*jalapão,*” “*mamoninha,*” “*raiz-de-teiú,*” “*peão-curador,*” “*peão-pajé,*” “*pião-caboclo,*” and “*pião-preto,*” among others [5, 8, 23]. There are also the following vernacular names for* J. gossypiifolia*: “*frailecillo,*” “*frailejón,*” “*purga de fraile*” (Colômbia); “*frailecillo*” (Costa Rica); “*frailecillo,*” “*San Juan Del Cabre,*” “*túatúa,*” “*tuba tuba*” (Cuba); “*baga*” (Malinké et Dioula); “*higuereta cimarrona,*” “*túatúa*” (Puerto Rico); “*túatúa*” (Santo Domingo); “*frailecillo,*” “*sibidigua,*” “*tuatuá*” (Venezuela); “*pignut,*” “*fignut,*” “*lapalapa,*” “*binidasugu,*” “*oluluidi,*” “*botuje red,*” “*botuge pupa*” (Nigeria); “*athalai,*” “*lal bherenda*” (Índia); “*parroty grass*” (Nicaragua); “*babatidjin*” (Africa); “*piñón,*” “*piñon-colorado,*” “*piñón negro,*” “*piñon-rojo,*” “*purga de huane*” (Spanish); “*herbe à mal de ventre,*” “*medicinier cathartique,*” “*medicinier sauvage*” (French); “*bellyachebuhs,*” “*bellyache bush,*” “*bellyache nettlespurge,*” “*black physicnut,*” “*purge nut,*” “*red fig-nut flower,*” “*wild cassada*” (English); “*babatidjin,*” “*balautandoiong,*” “*cassava marble,*” “*cotton-leaf physicnut,*” “*figus nut,*” “*kishka,*” “*lansi-lansinaan,*” “*médicinier bâtard,*” “*médicinier noir,*” “*médicinier rouge,*” “*quelite de fraile,*” “*sosori,*” “*tagumbau-a-nalabaga,*” “*tatua,*” “*tauataua,*” “*tautuba,*” “*tuatúa blanca,*” “*tuatúa morada,*” “*tubang morado,*” “*tuba sa buaia,*” “*tuba-tuba*” (Achanti); “*satamân*” (Bambara) [22, 24–31].

The complete taxonomy of the species is Eukaryota; Viridiplantae; Streptophyta; Streptophytina; Embryophyta; Tracheophyta; Euphyllophyta; Spermatophyta; Magnoliophyta; eudicotyledons; core eudicotyledons; rosids; fabids; Malpighiales; Euphorbiaceae; Crotonoideae; Jatropheae;* Jatropha; *and* Jatropha gossypiifolia *[32].* Adenoropium gossypiifolium *(L.) Pohl,* Manihot gossypiifolia *(L.) Crantz,* Adenoropium elegans *Pohl,* Jatropha elegans *Kl.,* Jatropha staphysagriifolia *Mill.,* Jatropha gossypifolia, *and* Jatropha gossipyifolia *are botanical synonymous of* J. gossypiifolia* species [8, 32–34].

*J. gossypiifolia* is a small shrub with dark green or more frequently purplish-red dark leaves, with 16–19 cm of length per 10–12.9 cm of width; they are alternate, palmate, and pubescent, with an acuminate apex, cordate base, and serrated margin. The flowers are unisexual, purple, and in cymose summits, with the calyx having five petals, which in male flowers may form a petaloid tube. The fruit is capsular, with three furrows, containing a dark seed with black spots [5, 35, 36]. Regarding the microscopic aspect of the plant leaves, some studies have shown key and important features for botanical identification of this species among other* Jatropha* species [21, 35–37].

## 4. Chemical Constituents

Various chemical constituents have been detected in extracts from different parts of* J. gossypiifolia*, the literature having reported, in general, the presence of fatty acids, sugars, alkaloids, amino acids, coumarins, steroids, flavonoids, lignans, proteins, saponins, tannins, and terpenoids, as can be seen in Table 1.

Accordingly reviewed by Zhang et al. [15], the main compounds isolated from* Jatropha* genus are the terpenoids. In fact, many of them were isolated from different parts of* J. gossypiifolia*. Another very important class from* J. gossypiifolia* is the lignoids, since a good number of them was already isolated and identified.

However, it is important to note that most of the phytochemical studies found in literature are not about isolation of compounds, but only about the phytochemical screening of the major classes through chemical qualitative reactions or more sensitive and specific methods such as thin layer chromatography (TLC). Relative to other* Jatropha* species, few studies have isolated chemical compounds from* J. gossypiifolia* (Table 2). In addition, up till now it is not clear which are the major bioactive compounds in the plant, since only a few studies were conducted by bioassay-guided isolation.

Additionally, to the best of our knowledge, there are no phytochemical studies regarding the use of water as solvent for the extraction of* J. gossypiifolia* constituents. This is important to note since popular use occurs more frequently with infusions or decoctions, and little is known about the constitution of this type of extract. In this context, it is important to conduct studies to evaluate the phytochemical constitution of these extracts. More commonly, the studies use solvents or mixtures of solvents with nonpolar characteristics, which could contribute to further characterization of nonpolar compounds, such as terpenoids and lignoids. Polar compounds such as flavonoids, tannins, and sugars are poorly described in the species so far, probably due to this fact.

## 5. Traditional Uses

Various medicinal properties for the species* J. gossypiifolia* are reported by traditional medicine, as shown in Table 3. Some properties related to* J. gossypiifolia* are also common to other species of the* Jatropha* genus [3, 9, 25], where human and veterinary uses are described. Different parts of this plant, such as leaves, stems, roots, seeds, and latex, are used in different forms of preparation (infusion, decoction, and maceration, among others), by different routes and forms (oral, topical, baths, etc.). The most frequent reports refer to its anti-inflammatory, antidiarrheal, antiophidian, analgesic, antipyretic, antimicrobial, healing, antianemic, antidiabetic, and antihemorrhagic activities, among many other examples [3, 5, 7, 9].

Some properties are attributed to specific parts of the plant, while others are assigned to different parts. Interestingly, in some cases certain uses may appear contradictory, such as antidiarrheal and laxative or its use as anticoagulant and antihemorrhagic. One hypothesis is that this difference may be related with the dose used, since, for example, the laxative effect is an effect commonly related with toxic events with this plant.

## 6. Pharmacological Activities

Despite the grand variety of popular uses and the data from* Jatropha* species,* J. gossypiifolia* has been scarcely studied regarding biological activities (Table 4). Studies showing the biological potential of aqueous extract are rare so far, which is important to be mentioned since the most popular use of this plant is as a tea (decoction or infusion). Among the main activities that have been studied the antihypertensive, anticancer, antimicrobial, healing, anti-inflammatory, and analgesic activities stand out.

### 6.1. Antihypertensive Action

Based on popular use of teas from* J. gossypiifolia* roots and aerial parts, the hypotensive and vasorelaxant effects of the ethanolic extract of aerial parts of the plant were tested by Abreu et al. [45]. The study revealed that the extract (125 and 250 mg/kg/day, over 4 weeks, by oral route in rats), in a dose-dependent manner, produced a reduction of systolic blood pressure in conscious normotensive animals. This hypotensive effect could be attributed to its vasorelaxant action, since it produced concentration-dependent relaxant effect in rat isolated endothelium-deprived mesenteric artery precontracted with norepinephrine or calcium. Moreover, it inhibited, in a concentration-dependent and noncompetitive manner, the contractile response induced by norepinephrine or CaCl_2_ in the same preparation [45].

### 6.2. Antimicrobial Action

The antibiotic activity of different extracts from* J. gossypiifolia* is frequently reported, as observed in Table 4. In general, some extent of antibacterial, antifungal, antiparasitic, and antiviral activity was observed. The only report of* J. gossypiifolia* isolated compound with antimicrobial activity is of the macrocyclic diterpene jatrophenone, which presented significant* in vitro* antibacterial activity against* Staphylococcus aureus* [41].

### 6.3. Anti-Inflammatory and/or Analgesic Action

Many important popular uses of* J. gossypiifolia* are related to inflammatory process. Bhagat et al. [28] showed that the methanolic extract of leaves of this species has significant systemic acute and chronic anti-inflammatory activity. The extract, at 500 and 1000 mg/kg oral doses, was able to inhibit the acute carrageenan-induced paw edema in rats and at 50 and 100 mg/kg oral doses inhibited the chronic cotton pellet-induced granuloma formation in rats. Additionally, the* J. gossypiifolia* leaf paste (0.5 and 1 mg/ear) showed significant reduction in TPA-induced local inflammatory changes in mouse ear edema model [28].

In another study, the anti-inflammatory and analgesic properties of the methanol and petrol ether extracts of aerial parts of* J. gossypiifolia* were demonstrated in mice [92]. At 100 and 200 mg/kg/day, during 7 days, by oral route, only the methanol extract presented significant analgesic activity in Eddy's hot plate and tail-flick models and anti-inflammatory activity in carrageenan-induced paw edema [92]. The anti-inflammatory activity of the bark from* J. gossypiifolia* (methanol and petrol ether extracts) was also demonstrated in carrageenan-induced paw edema in rats [115].

In a recent study, using the* in vitro* human red blood cell membrane stabilization method, Nagaharika et al. [118] suggested that ethanol and water extracts from* J. gossypiifolia *leaves have anti-inflammatory activity. According to the authors, since human red blood cell membranes are similar to the lysosomal membrane components, the prevention of hypotonicity-induced membrane lysis of these cells could be taken as a measure in estimating the anti-inflammatory property of compounds [118].

The analgesic activity of the methanol extract from the leaves of* J. gossypiifolia* was evaluated in acetic acid-induced writhing test in mice, where highly significant inhibition was seen of 67.56 and 65.14% at 200 and 400 mg/kg oral doses, respectively [111]. Similar results were observed in the methanolic extract from fruits [110].

### 6.4. Healing Action

The healing action of the ethanol crude extract of* J. gossypiifolia* (plant part not specified) was evaluated in suture healing of ventral abdominal wall of rats, through tensiometric measurement and macro- and microscopic aspect of postoperative period. The extract, which was administered by an intraperitoneal instillation of 100 mg/kg single dose in the peritoneal cavity, presented more intense adhesion on macroscopic examination and greater strain evaluation and vascular neoformation. However, a greater inflammatory process was also observed, and other histological parameters were similar to the control group, indicating that, in general, the extract presented poor wound healing properties in the used model [124].

Another study evaluated the healing action of the hydroethanolic crude extract from leaves of* J. gossypiifolia* in the healing process of sutures performed on the bladder of rats, and similar results were presented, although some improvement might have been observed in some parameters. In general, the authors concluded that no favorable healing effect was observed with the administration of single intraperitoneal dose of* J. gossypiifolia *L. [108]. In another study analyzing the morphological aspects of the healing process occurring in open skin lesions in rats under topical administration of raw extract from* J. gossypiifolia* (details about extract preparation and plant part not specified), the authors also observed an absence of healing action, although some histological improvement was shown [125].

However, studying the influence of* J. gossypiifolia* on the healing process of colonic anastomosis in rats, Servin et al. showed that the administration of 1 mL/kg single dose of the hydro alcoholic extract from aerial parts has beneficial effect on the healing process [122]. However, according to these authors, on the seventh day of the experiment, there was a decrease in the action of the extract, suggesting that the extract, in this experiment, was less active in later stages of healing process [122]. A plausible hypothesis, not raised by the authors, could be the fact that the extract was administered in a single dose, which may not have been sufficient to maintain the effect throughout the time of the experiment. Additionally, Vale et al. showed that the ethanolic extract from aerial parts of* J. gossypiifolia*, at single intraperitoneal dose of 200 mg/kg, favored the healing process of gastrorrhaphies and reduced the acute inflammatory reaction* in vivo* [123].

### 6.5. Hemostatic Action

The use of* J. gossypiifolia*, especially the latex, is widespread as a hemostatic agent for preventing bleeding disorders. The results of whole blood clotting time using Lee and White method and bleeding time using Ivy's method were significantly reduced when stem latex was introduced, suggesting procoagulant activity [101]. Regarding the possible mechanism of action, based on experiments that show the precipitating action of the latex upon bovine albumin, the authors suggest that the latex precipitates clotting factors thereby bringing the coagulation factors into close contact, and then the activation of coagulation cascade leads to the generation of thrombin and formation of a clot takes place in a matter of seconds when compared to the control experiment, which took minutes to complete coagulation [101]. It is important to emphasize that, to the best of our knowledge, this is the only study performed on human subjects.

### 6.6. Anticholinesterase Action

Based on the cholinergic hypothesis, acetylcholinesterase inhibitors are widely used to treat Alzheimer's disease.* J. gossypiifolia* presented an important anticholinesterase activity since the methanolic extract from leaves showed an IC_50_ of 0.05 mg/mL [117]. Another study showed that the lyophilized latex of the plant was able to inhibit time- and dose-dependently the acetylcholinesterase enzyme in nervous tissue of freshwater air breathing fish* Channa marulius* [116].

### 6.7. Antioxidant Action

The antioxidant activity of extracts from* J. gossypiifolia* was evaluated by Kharat et al. [55]. In this work the high content of phenols, tannins, and flavonoids in the leaves prompted the authors to evaluate the antioxidant activity of the leaves. DPPH free radical, ferric thiocyanate, and nitric oxide scavenging methods were used to analyze the antioxidant activity* in vitro* of methanol, ethyl acetate, and aqueous extracts, demonstrating positive results. The authors attributed the free radical scavenging activity to the presence of flavonoids [55]. On the other hand, a study showed that different extracts (petrol ether, chloroform, ethyl acetate, and* n*-butanol) from whole plant of* J. gossypiifolia* had only partial antioxidant activity in DPPH scavenging, total antioxidant capacity, and lipid peroxidation tests [48]. Among them, the ethyl acetate extract was the most active, which correlates positively with its higher content of phenolic compounds in comparison with the other extracts [48].

### 6.8. Contraceptive Action

Based on its popular use,* J. gossypiifolia* was assessed for its antifertility activity, as an alternative to oral contraceptive agents.* J. gossypiifolia* leaf extract, by oral route, altered the major hormones involved in estrous cycle regulation, indicating its antifertility effect on mice [121]. Evaluating other parameters (estrogenic and early abortifacient activities) the anti-infertility effect of the extract was once more demonstrated later [130].

### 6.9. Tocolytic Action

Based on the ethnopharmacological application of the plant as tocolytic remedy, the effects on calcium-evoked uterine smooth muscle contraction of the ethanolic extract and fractions were evaluated [129]. The crude extract and, to a higher extent, the chloroformic fraction reduced the calcium-evoked contractile response of the uterine smooth muscle, promoting a rightward displacement of calcium cumulative curves, as well as reducing the maximal contractions [129].

### 6.10. Antineoplasic Action

One of the most well-known pharmacological activities of* J. gossypiifolia* is its antineoplasic action, which is frequently associated with the content of lignoids and terpenoids. One of the first reports was made by Kupchan et al. [66], when the authors found that the ethanolic extract from roots, as well as the isolated diterpene jatrophone, exhibited significant inhibitory activity* in vitro* against cells derived from human carcinoma of the nasopharynx and lymphocytic leukemia P-388 and* in vivo* against four standard animal tumor systems, such as sarcoma 180, Lewis lung carcinoma, P-388 lymphocytic leukemia, and Walker 256 intramuscular carcinosarcoma [66]. Later, three new antitumor derivatives of jatrophone were isolated from petrol ether extracts from roots of* J. gossypiifolia*: 2*α*-hydroxyjatrophone, 2*β*-hydroxy-5,6-isojatrophone, and 2*β*-hydroxyjatrophone [64]. Recently, two other diterpenes with potent antineoplasic activity were isolated from* J. gossypiifolia*: falodone and abiodone. Falodone was isolated from methanol extract from roots and showed potent proliferation inhibitory activity against A-549 human cancer cell line [13]. Abiodone, a lathyrane diterpenoid compound, was isolated from* J. gossypiifolia* and presented potent anticancer activity [73].

### 6.11. Local Anesthetic Action

The local anesthetic action of* J. gossypiifolia* was evaluated by plexus anaesthesia in frogs [128]. The authors observed that the aqueous and methanol extract (plant part not specified) presented significant anesthetic action when compared to control group.

### 6.12. Neuropharmacological Action

The neuropharmacological action of the methanol extract of the leaves of* J. gossypiifolia* was evaluated by Apu et al. [111]. The authors observed that in hole cross test the extract at 200 and 400 mg/kg, by oral route, showed significant sedative effect in mice. In hole board test, the extract showed highly significant anxiolytic activity at a dose of 200 mg/kg, whereas the same activity was observed at 400 mg/kg dose in elevated plus-maze test [111]. Similar results were observed in the methanolic extract from fruits [110].

### 6.13. Antidiarrheal Action

Although it may seem contradictory as shown in Table 3,* J. gossypiifolia* species is popularly used both as purgative and as antidiarrheal remedy. However, in literature, there are interesting results about the antidiarrheal properties of different extracts of this species.

At 200 and 400 mg/kg oral doses in mice, the methanol extract of* J. gossypiifolia* leaves produced highly significant antidiarrheal activity upon castor oil-induced diarrhea, decreasing the mean number of stool and total weight of fecal output when compared to control group [111]. Similar results were observed in the methanolic extract from fruits [110].

Aiming to determine the possible action mechanism of* J. gossypiifolia* aerial parts ethanol extract as antidiarrheal agent, Silva et al. [119] have investigated the effect of this extract on intestinal transit velocity and on isolated rat jejunum. At 500, 1000, and 2000 mg/kg, by oral route in mice, the extract showed significant antispasmodic activity in mouse intestinal transit model when compared to control. At 0.5, 1.0, and 2.0 mg/mL, the crude extract inhibited* in vitro* the acetylcholine and calcium-induced contractions of isolated rat jejunum. The chloroform and aqueous fractions were obtained and it was observed that only the chloroform fraction of the extract had a calcium-antagonist effect, whereas both chloroformic and aqueous fractions had anticholinergic effect, suggesting that the antispasmodic effect of* J. gossypiifolia* may be due to a combination of anticholinergic and calcium-antagonist mechanisms [119].

### 6.14. Immunomodulatory Action

The immunomodulatory action of synthetic lignan compounds was evaluated by the assay of proliferation of mouse spleen cell* in vitro* and compared with petrol ether extract of whole plant of* J. gossypiifolia*, since it is a natural source of this kind of compound [127]. The authors showed that both synthetic and naturally occurring 1-phenylnaphthalene lignans could positively modulate the immunity of the host, since they significantly increased the proliferation of mouse spleen cell* in vitro* [127].

### 6.15. Hepatoprotective Action

Despite some studies having shown the hepatotoxic potential of* J. gossypiifolia*, a study was performed to analyze the possible hepatoprotective action of extracts of this plant in carbon tetrachloride-induced liver damage in rats [126]. In fact, the petrol ether, methanol, and water extracts from the aerial parts of* J. gossypiifolia* presented significant hepatoprotective action in this model, substantially restoring towards normalization the serum levels of serum glutamate oxaloacetate transaminase, serum glutamate pyruvate transaminase, serum alkaline phosphatase, total bilirubin, superoxide dismutase, and catalase [126]. The authors also discuss the close relationship between the hepatoprotective action observed and the possible antioxidant mechanism present in the extracts.

## 7. Other Actions and Biotechnological Applications

In addition to studies demonstrating scientific evidences of the pharmacological properties of* J. gossypiifolia*, several studies have demonstrated the potential of this species to obtain molecules with various applications, thus showing its multipurpose character.

Among the main applications described, the use of* J. gossypiifolia* seed oil for biodiesel production could be mentioned.* Jatropha* species has drawn the attention of researchers in recent years due to its emergence as a highly suitable feedstock plant for biodiesel production [11]. Among the species,* J. gossypiifolia*,* J. curcas,* and* J. pohliana* produce seeds with high oil content [11]. In a study investigating the potential of two plants of the* Jatropha* genus (including* J. gossypiifolia*), the authors observed that the studied physicochemical properties of the produced biodiesel are in the acceptable range for use as biodiesel in diesel engines, showing a promising economic exploitation of these raw materials [131].

Studies have shown the potential of the species for the development of new tools for biochemical analysis. A recent study showed that the diluted fresh latex* J. gossypiifolia* can be used as precipitating agent for biochemical determination of proteins in plasma, urine, and cerebrospinal fluid, with values comparable to those obtained from the conventional protein precipitants sodium tungstate and trichloroacetic acid [24]. According to the authors, the precipitating potential could be related to the capacity of the latex to form clots when applied to a bleeding sore or wound when it is used in folk medicine [24]. Another study showed the potentiality of the juice extracted from the fresh leaves of* J. gossypiifolia* as an anticoagulant for haematological analyses [86]. 0.1 mL of extract per mL of blood proved to be suitable for obtaining plasmas for biochemical analysis comparable with conventional anticoagulants [86]. However, the authors emphasize that the extract must be purified to remove interfering substances for it to be perfectly suitable for biochemical analysis [86].

Some studies have demonstrated the potentiality of* J. gossypiifolia* as a source of pesticide biomolecules. Bullangpoti et al. [49] isolated ricinine from the ethyl acetate extract from senescent leaves, the main compound responsible for the toxicity of the crude extract in* Spodoptera exigua* larvae, thus demonstrating that it could be an alternative choice to chemical insecticides. In another study, Bullangpoti et al. [132] showed that the ethanol extract of* J. gossypiifolia* in association with the ethanol extract of* Melia azedarach *was toxic and inhibited some enzymes from* Spodoptera frugiperda* larvae, demonstrating once more the potentiality of the species as insecticide agent. Calatayud et al. [56] showed the presence of proteins of about 100 kDa with toxic activity upon* Phenacoccus herreni, *another type of insect. In this work, the authors performed a strategy of extraction that eliminated nonprotein compounds, being able to demonstrate the potential of the species to obtain insecticidal proteins [56]. Leaf extract of* J. gossypiifolia* reduced the fecundity and egg viability against stored product insect pests* Tribolium castaneum* [133].

The potential molluscicidal activity of* J. gossypiifolia* has also been evaluated as an alternative mode of prevention of schistosomiasis. Sukumaran et al. [134] showed that the methanol and* n*-butanol extracts from unripened seeds of* J. gossypiifolia* was toxic against eggs and adults of two species of freshwater snails,* Lymnaea luteola* and* Indoplanorbis exustus*. The results indicated that* n*-butanol extract was the most effective and that the eggs were more susceptible than adults [134].

## 8. Toxicology

Species of* Jatropha* are notably known for their toxic potential [135, 136]. This toxicity is related primarily to latex and seeds. The latex is released from the aerial parts of the plant by mechanical injury and it is extremely caustic and irritating to skin and mucous membranes. The seeds are rich in toxalbumins that cause agglutination and hemolysis to erythrocytes as well as damage to other cell types and contain a lipoid resin complex that can cause dermatitis [3, 12, 135]. The symptomatology consists, in general, of gastrointestinal disorders (abdominal pain, nausea, vomiting, and diarrhea). Additionally, the clinical course can bring cardiovascular, neurological, and renal complications [136]. Cases of poisoning in humans usually occur by eating fruit and seeds because of its similarity to edible chestnuts [136].

Some toxicological studies have demonstrated the toxic properties of* J. gossypiifolia*, while others show the absence of toxicity. However, it is important to observe the models used, doses administrated, and types of extract employed (solvent and plant part), among other aspects, to make the proper conclusions about the toxicity.

The study of experimental poisoning in sheep showed that the intake of fresh plant leaves in a single dose of 40 g/kg was lethal to these animals [137]. The clinical and pathological picture in the experimental sheep was characterized by digestive, lung, and heart disturbances and also by slight regressive changes evidenced in hepatic and renal histological examinations [137]. However, as observed by Mariz et al. [7], it is important to note that the medicinal use of the plant is rarely* in natura*, but instead by different preparations, such as infusions or decoctions, sometimes of the dried material, which could inactivate the possible toxic components. However, this is only a hypothesis, and so the toxicity of extracts from leaves cannot be discarded.

One of the first studies relating the identification of the constituents responsible for the toxic effects of the* Jatropha* species was published by Adolf et al. [69]. In this work, by a bioguided isolation, the irritant polyunsaturated ester 12-deoxy-16-hydroxylphorbol was isolated from the ether extract from the seeds of* J. gossypiifolia* by countercurrent chromatography [69]. The irritant activity was visualized in mouse ear after 24 h of the application of the fractions and isolated compounds [69].

The* in vitro* cytotoxicity assay using brine shrimp larvae test revealed that ethanol and methanol extracts (plant organ unspecified) showed low toxicity [138]. An earlier study showed that the water and ethyl acetate fraction of a methanol extract from aerial parts of* J. gossypiifolia* did not present toxicity against the same organisms [114].

A study performed in Wistar rats evaluated the toxicity of the ethanolic root extract of* J. gossypiifolia* at 10, 20, and 30 mg/kg by oral route [139]. The authors observed that the extract was toxic to the kidney and caused increased urea retention in the blood, as observed by histological studies and biochemical analysis of blood [139].

A preclinical toxicological assessment of the crude ethanol extract from* J. gossypiifolia* leaves showed that the extract presents relatively low oral acute toxicity in Wistar rats [18, 19]. Rats treated with single doses of 1.2–5.0 g/kg by oral route were observed for 14 days, and the most important signs of toxicity were ptosis, reduction of body weight, and hind limb paralysis. Other significant alterations occurred only in males treated with 5.0 g/kg dose: increase in creatinine, aspartate aminotransferase, sodium and potassium seric levels, reduction of urea and albumin, leucopenia and small alteration in color, and consistency of viscera. The median lethal dose (LD_50_) was higher than 4.0 g/kg for males and higher than 5.0 g/kg for females [19]. In the histopathological evaluation some alteration was observed in liver and lung only at 5.0 g/kg, suggesting the relatively low toxicity of the extract [18]. However, in the chronic toxicological study (thirteen weeks of treatment), this extract showed significant oral chronic toxicity in rats [17]. The most significant toxic signs indicated a reduction of the activity in the central nervous system and digestive disturbances. The histopathological analysis revealed hepatotoxicity and pulmonary damages. The lethality was 46.6% and 13.3% among males and females under the higher tested dose (405 mg/kg), respectively [17]. Based on this, Mariz et al. [7] discussed that the development of herbal medicine based on this species needs to prioritize the chemical refinement of the crude extracts to obtain less toxic fractions, which should be tested for their safety and therapeutic efficacy.

Another study, on the other hand, evaluating the oral acute toxicity of the aqueous and ethanol extracts from leaves of* J. gossypiifolia*, did not show any sign of toxicity in up to 2 g/kg in rats, enabling the authors to conclude that this extract could be considered safe [118]. This is an interesting result since in most cases the plant is used popularly as tea (aqueous extract).

The toxicity of the stem latex of* J. gossypiifolia* was studied in Wistar rats by applying different doses of crude latex on incised skin daily for 18 days, based on the popular use of the latex as hemostatic agent in skin lesions [140]. The authors observed that the application of the latex did not produce any significant difference in results of biochemical and hematological parameters obtained from the control and experimental animals, leading to the conclusion that the stem latex has no harmful effects [140].

## 9. Conclusions

As demonstrated by this review,* J. gossypiifolia* presents an important potential for the generation of pharmacological and/or biotechnological products, based on popular uses and biological studies scientifically showing its properties. However, regarding specifically its medicinal properties, further studies are still necessary to assay important folk uses of the species and characterize the major compounds responsible for the bioactivity. Thus, studies of bioprospecting could prioritize this species, since many popular uses for various medical purposes are reported, demonstrating a great potential to originate bioactive molecules with pharmacological relevance. Furthermore, future phytochemical studies of this plant are important to obtain the best knowledge of the chemical composition of different extracts of the plant, in order to recognize the really important compounds in the pharmacological actions, aspiring to the chemical refinement of the products to eliminate the eventual toxic effects that could reduce the medicinal value of the species. In conclusion, the data presented in this review could provide insights for future research aimed at both ethnopharmacological validation of the popular use of* J. gossypiifolia* and its exploration as a new source of bioactive molecules for herbal drugs and/or bioactive natural products for potential application in complementary and alternative medicine.

## Figures and Tables

**Table 1:** Chemical constituents of *Jatropha gossypiifolia* L. described in the literature.

Plant part	Classification	Compound	Extract type and/or preparation	Reference
Whole plant	Coumarin-lignoids	Propacin	Isolated from dichloromethane : methanol (1 : 1, v/v) extract after successive column chromatography on silica gel	[38]
		Venkatasin	Not specified*	[39]
	Diterpenes	Citlalitrione	Isolated from dichloromethane : methanol (1 : 1, v/v) extract after successive column chromatography on silica gel	[40]
		Jatrophenone	Isolated from dichloromethane : methanol (1 : 1, v/v) extract after successive column chromatography on silica gel	[41]

Stem, roots, and seeds	Coumarin-lignoids	Arylnaphthalene lignan	Isolated from petrol ether extract after successive column chromatography on silica gel	[42]
		Gadain	Isolated from petrol ether extract after successive column chromatography on silica gel	[43]
		Jatrophan	Isolated from petrol ether extract	[44]

Aerial parts	Flavonoids	—	Detected by phytochemical screening reactions of ethanol extract	[19, 45]
	Lignans	Gossypifan	Isolated from petrol ether extract after successive column chromatography on silica gel	[46]
		Gossypiline	Isolated from dichloromethane : methanol (1 : 1, v/v) extract after successive column chromatography on silica gel	[47]
	Phenols	—	Quantitative analysis showed that the petrol ether, chloroform, ethyl acetate, and *n*-butanol extracts presented, respectively, 45.0 ± 1.0, 106.0 ± 2.3, 296.0 ± 3.5, and 128.5 ± 1.1 mg of gallic acid equivalents/g of crude extract	[48]
	Steroids	—	Detected by phytochemical screening reactions of ethanol extract	[19, 45]
	Tannins	—	Detected by phytochemical screening reactions of ethanol extract	[23, 45]
	Triterpenoids	—	Detected by phytochemical screening reactions of ethanol extract	[45]

Leaves	Alkaloids	Ricinine	Compound isolated from ethyl acetate extract from senescent leaves	[49]
		—	Detected by phytochemical screening reactions of chloroform and methanol extracts	[50]
		—	Quantitative analysis showed 2.81% on leaves	[51]
		—	Not specified	[30]
	Cardiac glycosides	—	Identified on leaves by qualitative phytochemical screening reactions	[52]
	Flavonoids	Apigenin	Identified in ether fraction from ethanol extract	[53]
		Isovitexin	Identified in ethyl acetate and methyl ethyl ketone fractions from ethanol extract	[53]
		Orientin/isoorientin	Isomers identified in different types of extracts from leaves	[54]
		Schaftoside/isoschaftoside	Isomers identified in different types of extracts from leaves	[54]
		Vitexin	Identified in ethyl acetate fraction from ethanol extract	[53]
		Vitexin/isovitexin	Isomers identified in different types of extracts from leaves	[54]
		—	Identified on leaves by qualitative phytochemical screening reactions	[52]
		—	Quantitative analysis showed 7.4% on leaves	[55]
		—	Quantitative analysis showed 2.41% on leaves	[51]
	Phenols	—	Quantitative analysis showed 8.6% on leaves	[55]
		—	Quantitative analysis showed 0.26% on leaves	[51]
	Phlobotannins	—	Detected by phytochemical screening reactions of chloroform and methanol extracts	[50]
	Proteins	—	Identified on leaves by qualitative phytochemical screening reactions	[52]
		—	Leaves obtained by micropropagation were macerated in liquid nitrogen and extracted at 4°C for 6 h with 0.1 M NaCl. The material was centrifuged and the limpid supernatant was dialyzed against water at low temperature in a cellulose membrane to remove nonprotein compound with molecular mass below 3.5 kDa	[56]
	Reducing sugars	—	Identified on leaves by qualitative phytochemical screening reactions	[52]
	Saponins	—	Identified on leaves by qualitative phytochemical screening reactions	[52]
		—	Quantitative analysis showed 4.15% on leaves	[51]
	Steroids	—	Identified on leaves by qualitative phytochemical screening reactions	[52]
	Tannins	—	Detected by phytochemical screening reactions of methanol extract	[50]
		—	Detected on leaves by qualitative phytochemical screening reactions	[52]
		—	Quantitative analysis showed 5.14% on leaves	[51]
	Terpenoids	—	Detected on leaves by qualitative phytochemical screening reactions	[52]
	Triterpenes	(2*α*, 13*α*, 14*β*, 20S)-2,24,25-Trihydroxylanost-7-en-3-one	Isolated from the ethanol extract after successive partitions procedures and column chromatography on silica gel and preparative TLC	[57]
		(13*α*, 14*β*, 20S)-2,24,25-Trihydroxylanosta-1,7-dien-3-one	Isolated from the ethanol extract after successive partition procedures and column chromatography on silica gel and preparative TLC	[57]

Stems	Alkaloids	—	Quantitative analysis showed 2.16% of alkaloid on stems	[51]
	Coumarin-lignoids	4′-*O*-Demethyl retrochinensin	Not specified	[58]
		Cleomiscosin A	Compound isolated from ethyl acetate fraction stems after successive column chromatography on silica gel	[59]
		Gossypidien	Compound isolated from hexane extract from dried stems after successive column chromatography on silica gel	[60]
		Isogadain	Not specified*	[61]
		Jatrodien	Compound isolated from petrol ether extract after successive column chromatography on silica gel	[62]
		Prasanthaline	Not specified*	[63]
	Flavonoids	—	Quantitative analysis showed 1.2% on stems	[51]
	Phenols	—	Quantitative analysis showed 0.13% on stems	[51]
	Saponins	—	Quantitative analysis showed 2.18% on stems	[51]
	Tannins	—	Quantitative analysis showed 1.36% on stems	[51]

Roots	Alkaloids	—	Quantitative analysis showed 1.6% on roots	[51]
	Diterpenes	2*α*-Hydroxyjatrophone	Isolated from petrol ether extract after successive column chromatography on silica gel	[64]
		2*β*-Hydroxy-5,6-isojatrophone	Isolated from petrol ether extract after successive column chromatography on silica gel	[64]
		2*β*-Hydroxyjatrophone	Isolated from petrol ether extract after successive column chromatography on silica gel	[64]
		Citlalitrione	Isolated from petrol ether fraction from the methanol extract after successive column chromatography on silica gel	[13]
		Falodone	Isolated from petrol ether fraction from the methanol extract after successive column chromatography on silica gel	[13]
		Jatropholone A	Not specified*	[65]
		Jatropholone B	Not specified*	[65]
		Jatrophone	Isolated from ethanol extract	[66]
	Flavonoids	—	Quantitative analysis showed 1.75% on roots	[51]
	Phenols	—	Quantitative analysis showed 0.24% on roots	[51]
	Saponins	—	Quantitative analysis showed 2.83% on roots	[51]
	Tannins	—	Quantitative analysis showed 2.73% on roots	[51]

Seeds	Alkaloids	—	Quantitative analysis showed 2.36% on seeds	[51]
	Amino acids	—	Not specified*	[67]
	Carbohydrates	—	Quantitative analysis showed 30.32% on seeds	[68]
		—	Not specified*	[67]
	Esters	12-Deoxy-16-hydroxylphorbol	Isolated from hydrophilic fraction from the ether extract, by countercurrent chromatography	[69]
	Fatty acids	Arachidic acid	Identified in petrol ether extract	[68, 70]
		Caprilic acid	Identified in petrol ether extract	[68, 70]
		Lauric acid	Identified in petrol ether extract	[68, 70]
		Lignoceric acid	Identified in petrol ether extract	[68, 70]
		Linoleic acid	Identified in petrol ether extract	[68, 70]
		Myristic acid	Identified in petrol ether extract	[68, 70]
		Oleic acid	Identified in petrol ether extract	[68, 70]
		Palmitic acid	Identified in petrol ether extract	[68, 70]
		Palmitoleic acid	Identified in petrol ether extract	[68, 70]
		Ricinoleic acid	Identified in petrol ether extract	[68, 70]
		Stearic acid	Identified in petrol ether extract	[68, 70]
		Vernolic acid	Identified in petrol ether extract	[68, 70]
	Fibers	—	Quantitative analysis showed 9.25% on seeds	[68]
	Flavonoids	—	Quantitative analysis showed 2.26% on seeds	[51]
	Phenols	—	Quantitative analysis showed 0.18% on seeds	[51]
	Proteins	—	Quantitative analysis showed 13.40% on seeds	[68]
	Saponins	—	Quantitative analysis showed 2.37 on seeds	[51]
			Quantitative analysis showed 6 g/kg on seeds	[68]
	Tannins	—	Quantitative analysis showed 3.52% on seeds	[51]

Latex	Proteins	Cyclogossine A	Not specified	[71]
		Cyclogossine B	Isolated from ethyl acetate extract by gel filtration column chromatography	[20]

Not specified	Alkaloids	Imidazole alkaloid	Isolated from the plant exudates*	[72]
		Piperidine	Isolated from the plant exudates*	[72]
	Diterpenoids	Abiodone	Not specified*	[73]

*The complete version of the paper was not accessible, so the information was obtained from its abstract.

**Table 2:** Main isolated compounds from *Jatropha gossypiifolia* L. described in the literature.

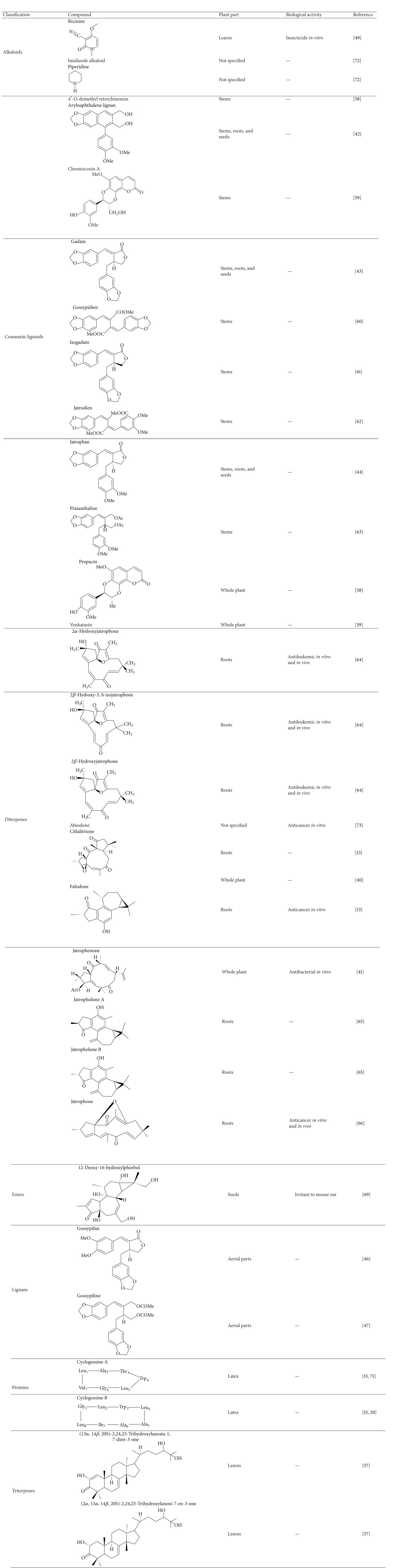

**Table 3:** Popular medicinal uses of *Jatropha gossypiifolia* L. described in the literature.

Plant part	Popular use	Preparation and/or mode of use	Reference
Whole plant	Analgesic (headache)	Leaves anointed with “*Sebo de Holanda*” (mutton tallow) and heated in the fire are used as compress for headaches	[5]
	Analgesic (toothache)	Not specified	[3]
	Antimicrobial	Not specified	[3]
	Antipyretic	Decoction	[20]
	Dyscrasia	Not specified	[3]
	Dysphonia	Not specified	[3]
	Wound healing	Not specified	[74, 75]

Aerial parts	Antianemic (malaria treatment)	Decoction, used by oral route	[76]

Leaves	Abscess	Bath	[77]
	Alopecia	Ash leaves	[25]
	Analgesic (eye pain)	Not specified	[78]
	Analgesic (headache)	Not specified	[78]
	Analgesic (headache and otitis)	Not specified	[79]
	Analgesic (pain in general)	Decoction or infusion	[80]
	Analgesic (toothache)	Decoction or infusion	[80]
	Antianemic	Decoction	[81]
		Decoction by oral route	[82]
	Anticancer	Ash of leaves	[25]
		Decoction of the association of leaves of *J. gossypiifolia* with leaves of *Petiveria alliacea* and aerial parts of *Stachytarpheta jamaicensis*, by oral route	[29]
		Not specified	[3]
	Anticonvulsivant	Not specified	[83]
	Antidiabetic	Decoction	[84, 85]
		Decoction by oral route	[30]
	Antidiarrheal	Decoction by oral route	[30]
		Not specified	[3]
	Antihemorrhagic	Decoction by oral route	[30]
		Fresh crushed leaves are used in cases of cutaneous and nasal bleeding	[86]
	Anti-infective	Decoction by oral route	[30]
		Not specified	[87, 88]
	Anti-inflammatory	Not specified	[78]
	Antipyretic	Decoction	[81]
		“Tea”	[5]
		Not specified	[88]
	Antiseptic	Bath prepared from the leaves	[5]
	Antithrombotic	Decoction or infusion	[80]
	Antiulcerogenic	Decoction by oral route	[30]
		Leaf juice	[89]
	Boils	Application of the pounded leaves	[90]
	Burns	Ash of leaves	[25]
		Used in association with seeds of *Gossypium arboreum*, sugar, honey bee, and fat of ram, prepared by grinding, applied topically	[29]
	Contraceptive and oxitotoxic	Not specified	[79]
	Depurative	Squeezed, the juice obtained is drunk	[91]
	Detoxificant	Not specified	[92]
	Eczema	Ash of leaves	[25]
	Emetic	Squeezed, the juice obtained is drunk	[91]
	Gastrointestinal disorders	Not specified	[79]
	Gingivitis	Leaf juice	[89]
	Gonorrhoea	Ash of leaves	[25]
	Healing	Bath prepared from the leaves	[5]
		Decoction	[30]
		Decoction or infusion	[80]
	Hemorrhoids	Used in association with leaves of *Nicotiana tabacum* and copper sulphate, boiled in water, and used as steam directed at the anal region	[93]
	Hemostatic	Decoction or infusion	[80]
	Hepatitis	Not specified	[12]
	Itching skin	Application of the pounded leaves	[90]
	Leprosy	Leaf juice	[89]
	Malaria	Decoction	[81]
		Decoction by oral route	[82]
		Used in association with leaves of *Azadirachta indica* and *Combretum* sp., boiled, for steam baths and by oral route	[94]
		Used in association with leaves of *Combretum ghasalense* and whole plant of *Ocimum canum*, by oral route or for steam baths	[94]
	Mastitis	Pounded leaves applied on swollen breasts	[90]
	Mycosis	Ash of leaves	[25]
	Psychoactive	Not specified	[79]
	Purgative	Not specified	[3, 88]
	Rheumatism	Ash of leaves	[25]
	Scabies	Ash of leaves	[25]
	Skin diseases	Not specified	[3]
	Stomachic	Decoction by oral route	[30]
		Not specified	[88, 92]
	Syphilis	Ash of leaves	[25]
	Thrush (oral candidiasis)	Ash of leaves	[25]
	Treatment of “cultural syndromes,” “*derrame,” “quebrante,” “espante,” “vento-caído,” “panema,” “doença-do-ar,” “mãe-do-corpo*”	Not specified	[79]
	Vaginal infection	Slightly boiled, used as vaginal wash	[91]
	Veneral diseases	Not specified	[92]
	Vermifuge	Ash of leaves	[25]
	Vertigo	Not specified	[3]
	Wounds and rashes	Bath of the leaves	[24]
		Decoction by oral route	[30]
		Decoction used as baths for cleaning wounds in dogs	[95]
	Wound disinfectant	Slightly boiled, used as wound wash	[91]

Stem	Analgesic (toothache)	Not specified	[96]
	Antianemic	Decoction by oral route	[82]
	Anticancer	Decoction by oral or topical route	[26]
	Emmenagogue	Decoction of barks	[70, 92]
	Malaria	Decoction by oral route	[82]
	Rheumatism	Not specified	[77]
	Thick blood	Not specified	[77]

Roots	Anticancer	Decoction by oral or topical route	[26]
		Root bark used for cancer of the lungs	[73]
	Anticonvulsivant	Not specified	[83]
	Antidiarrheal	Not specified	[89]
	Antimicrobial	Root bark used in bacterial infections	[73]
	Impotence	Decoction of the association of roots of *J. gossypiifolia*, *Chiococca alba, Citrus aurantifolia, Desmodium canum, Roystonea regia, Senna occidentalis, Stachytarpheta jamaicensis,* and* Waltheria indica* with the whole plant of *Commelina erecta, Cyperus rotundus,* and sugar, by oral route	[29]
	Leprosy	Not specified	[3, 92]
	Snakebites	Not specified	[22, 92, 97, 98]
	Urinary pain	Not specified	[92]
	Uterus diseases	Decoction by oral route	[99, 100]

Seeds	Analgesic (body pain)	Not specified	[101]
	Analgesic (headache)	Not specified	[79]
	Antigripal	Used in strong colds	[5]
	Antihemorrhagic	Not specified	[9]
	Antiulcerogenic	Seed oil	[3]
	Contraceptive and oxitotoxic	Not specified	[79]
	Depurative	Not specified	[91]
	Emetic	Not specified	[70, 91, 101]
	Gastrointestinal disorders	Not specified	[79]
	Leprosy	Seed oil	[3]
	Mycosis	Seed oil	[3]
	Psychoactive	Not specified	[79]
	Purgative	Not specified	[3, 9, 101, 102]
	Treatment of “cultural syndromes,” “*derrame,” “quebrante,” “espante,” “vento-caído,” “panema,” “doença-do-ar,” “mãe-do-corpo*”	Not specified	[79]
	Vaginal infection	Slightly boiled, used as vaginal wash	[91]
	Wound infection	Slightly boiled, used as wound wash	[91]

Fruits	Analgesic	Massaging pregnant women's bellies with tea or garrafada* when they are in pain	[77]
	Analgesic (headache)	Tea or garrafada*	[77]
	Analgesic (toothache)	Tea or garrafada*	[77]
	Laxative	Ingestion *in natura* of the powder fruit	[102]
	Numbness after bug stings	Tea or garrafada*	[77]

Latex	Alopecia	Not specified	[25]
	Analgesic (eye pain)	Not specified	[78]
	Analgesic (pain in general)	Drink or massage the affected area with latex	[80]
	Analgesic (toothache)	Cotton soaked with latex kept in contact with the sore tooth	[103]
		Drink or massage the affected area with latex	[80]
	Anticancer	Not specified	[25]
	Antihemorrhagic	Not specified	[9, 24, 86, 95]
	Antithrombotic	Oral route	[80]
	Antiulcerogenic	Not specified	[20, 89]
	Bite of venomous animals	Application of fresh latex at the affected site	[5]
	Diuretic	A few drops of fresh latex in water	[6]
	Eczema	Not specified	[25]
	Gingivitis	Not specified	[89]
	Gonorrhea	Not specified	[25]
	Hemostatic	Not specified	[25, 80]
	Infected wounds	Application of fresh latex at the affected site	[5, 20]
	Leprosy	Not specified	[89]
	Mycosis	Not specified	[25]
	Purgative	A few drops of fresh latex in water	[6]
		Not specified	[9]
	Rheumatism	Not specified	[25]
	Scabies	Not specified	[25]
	Skin burns	Application of fresh latex at the affected site	[104]
		Not specified	[25]
	Stop of itching of cuts and scratches	Not specified	[95]
	Syphilis	Not specified	[25]
	Thrush (oral candidiasis)	Not specified	[25]
	Vermifuge	Not specified	[25]
	Wound healing	Application of latex at the affected site	[5]
		Drink or massage the affected site with latex	[80]
		Not specified	[74, 75]

Resin	Toothache	Toothpowder	[27]
	Wounds in lips and tongue	Topical application	[27]

Oil	Arthritis	Applied locally	[89]
	Purgative	Not specified	[89]
	Skin disease	Applied locally	[89]

Not specified	Alopecia	Tea applied locally in dogs	[105]
	Analgesic	Not specified	[13]
		Poultices	[95]
	Anticancer	Not specified	[13, 66]
	Antidiarrheal	Not specified	[28, 45, 106]
	Antihypertensive	Not specified	[45]
	Anti-inflammatory	Not specified	[13, 28]
	Antipyretic	Not specified	[28]
	Antiseptic	Not specified	[45]
	Antiulcerogenic	Not specified	[28]
	Coughs and colds	Bark juice (4 spoonfuls, 3 times a day) by oral route	[107]
	Detoxication	Not specified	[28]
	Diuretic	Not specified	[45]
	Eczema	Not specified	[28]
	Gum infection	Not specified	[28]
	Healing	Not specified	[45, 108]
	Hydropsy	Not specified	[5]
	Leprosy	Not specified	[28]
	Obstructions of the abdominal tract	Not specified	[5]
	Purgative	Not specified	[5]
	Regulate menses	Not specified	[109]
	Rheumatism	Not specified	[5]
	Snake and scorpion bites	Not specified	[3, 22]
	Stomach pain	Not specified	[28]
	Venereal diseases	Not specified	[28]
	Wounds	Poultices	[95]
		Used as bath	[28]

*Garrafada: preparation done by macerating plant parts in alcohol or hydroalcoholic mediums, in general, brandies.

**Table 4:** Pharmacological studies of *Jatropha gossypiifolia* L. described in the literature.

Pharmacological activity	Plant part	Extract/compounds	Detail	Reference
Analgesic	Aerial parts	Methanol and petrol ether extracts	At 100 and 200 mg/kg/day, over 7 days, by oral route in mice, only the methanol extract presented significant analgesic activity in Eddy's hot plate and tail-flack models	[92]
	Fruits	Methanol extract	At 200 and 400 mg/kg, by oral route in mice, highly significantly inhibited the writhing responses induced by acetic acid	[110]
	Leaves	Methanol extract	At 200 and 400 mg/kg, by oral route in mice, significantly inhibited the writhing responses induced by acetic acid	[111]

Antibacterial	Latex	Crude latex	At 100 *μ*L volume inhibited *in vitro Listeria monocytogenes, Salmonella tyhimurium*, *Salmonella typhi*, and *Staphylococcus aureus *	[112]
	Latex	Not specified	Presented bactericidal effect *in vitro* against *Shigella dysenteriae* and Staphylococcus aureus*	[113]
	Leaves	Fractions obtained by sequential extraction of the vegetal material with petrol ether, benzene, chloroform, acetone, ethanol, methanol, and water	Petrol ether fraction was inactive against *Escherichia coli* and *Bacillus subtilis*. Benzene fraction was the most active, against both microorganisms. Chloroform and methanol fractions were active only against *Bacillus subtilis*. Acetone and ethanol fractions were active only against *Escherichia coli. *Aqueous fraction was active against both microorganisms, although to a much lesser degree than the other fractions.	[52]
		Methanol, chloroform, and water extracts	All extracts were active *in vitro* against Shigella dysenteriae*	[113]
		Petrol ether and ethyl acetate fractions from ethanol : dichloromethane (1 : 1, v/v) extract	The petrol ether fraction (1 mg/mL) inhibited *in vitro Pseudomonas aeruginosa*, *Staphylococcus epidermidis,* and *Salmonella typhimurium. *The ethyl acetate fraction (1 mg/mL) was active against *Staphylococcus aureus *	[87]
	Whole plant	Jatrophenone	Presented *in vitro* antibacterial activity against *Staphylococcus aureus *comparable to penicillin	[41]

Antibacterial and antifungal	Aerial parts	Water and ethyl acetate fractions from methanol extract	Both fractions, at 1 mg, did not produce zones of inhibition for *Escherichia coli*, *Staphylococcus aureus*, *Saccharomyces cerevisiae,*nor *Candida albicans *	[114]
	Leaves	Chloroform extract	Presented antibacterial activity against *Salmonella typhi*, *Pseudomonas aeruginosa,*,and *Staphylococcus aureus* and antifungal activity against *Candida albicans. *Did not produce inhibition zones against *Escherichia coli, Bacillus subtilis, Proteus mirabilis, Corynebacterium diptheriae, Shigella dysenteriae,*and *Streptococcus penumoniae *	[50]
	Leaves	Dichloromethane : methanol (1 : 1, v/v) extract	At 0.5 and 1 mg/mL, showed significant antibacterial activity *in vitro* against *Bacillus cereus* var *mycoides*, *Bacillus pumilus*, *Bacillus subtilis*, *Bordetella bronchiseptica*, *Micrococcus luteus*, *Staphylococcus aureus, Staphylococcus epidermidis, Klebsiella pneumoniae,* and *Streptococcus faecalis* and antifungal activity *in vitro* against *Candida albicans *	[88]
		Methanol extract	Presented antibacterial activity against *Salmonella typhi*, *Pseudomonas aeruginosa,* and *Staphylococcus aureus* and antifungal activity against *Candida albicans. *Did not produce inhibition zones against *Escherichia coli, Bacillus subtilis, Proteus mirabilis, Corynebacterium diptheriae, Shigella dysenteriae,*and *Streptococcus penumoniae *	[50]
	Not specified	Extracts obtained by sequential extraction of the vegetal material with *n*-hexane, chloroform, acetone, methanol, and water	*n*-Hexane extract had inhibitory activity *in vitro* against *Escherichia coli*, *Salmonella typhi*, *Pseudomonas aeruginosa*, *Bacillus cereus*, *Klebsiella aerogenes*, and *Candida albicans *but was inactive against *Shiguella boydi*, *Aspergillus fumigatus*, *Aspergillus flavus,* and *Aspergillus niger*. Chloroform extract inhibited *in vitro Salmonella typhi*, *Pseudomonas aeruginosa*, *Bacillus cereus,*and *Candida albicans *but was inactive against *Escherichia coli, Staphylococcus aureus, Shiguella boydi, Aspergillus fumigatus, Aspergillus flavus*, and *Aspergillus niger. *Acetone extract inhibited *in vitro Escherichia coli, Pseudomonas aeruginosa, Staphylococcus aureus, Klebsiella aerogenes, Proteus vulgaris,*and *Candida albicans *but was inactive against *Salmonella typhi, Aspergillus fumigatus, Aspergillus flavus,*and* Aspergillus niger. *Methanol extract inhibited *in vitro Escherichia coli, Salmonella typhi, Pseudomonas aeruginosa, Staphylococcus aureus, Bacillus cereus,*and *Candida albicans *but was inactive against *Aspergillus fumigatus, Aspergillus flavus,*and *Aspergillus niger. *Water extract was active *in vitro* against *Escherichia coli, Salmonella typhi, Pseudomonas aeruginosa, Staphylococcus aureus, Bacillus cereus, Klebsiella aerogenes, Proteus vulgaris,*and *Candida albicans *but was inactive against* Aspergillus fumigatus, Aspergillus flavus,* and *Aspergillus niger *	[89]
		Methanol and petrol ether extracts from bark	At 200 *μ*g/100 *μ*L, only the methanol extract showed *in vitro* antibacterial activity upon *Staphylococcus aureus*, *Streptococcus pyogenes,* and *Escherichia coli* and antifungal activity upon *Aspergillus niger*, *Candida albicans*, *Penicillium notatum,* and *Saccharomyces cerevisiae *	[115]

Anticholinesterase	Latex	Lyophilized latex	Inhibited time- and dose-dependently the acetylcholinesterase enzyme in nervous tissue of freshwater air breathing fish *Channa marulius *	[116]
	Leaves	Fractions obtained by sequential extraction of the vegetal material with ethyl acetate and methanol	At 2 mg/mL concentration, the ethyl acetate and methanol fractions presented inhibitory activities *in vitro* of 71 and 100%. The methanol fraction presented IC_50_ of 0.05 mg/mL	[117]

Antidiarrheal	Fruits	Methanol extract	At 200 and 400 mg/kg, by oral route in mice, inhibited the castor oil induced diarrhea	[110]
	Leaves	Methanol extract	At 200 and 400 mg/kg, by oral route in mice, inhibited the castor oil induced diarrhea	[111]

Antifungal (antidermatophytic fungi)	Aerial parts	Water and ethyl acetate fractions from methanol extract	The minimal concentration producing 75% of inhibition or higher against *Microsporus canis,* for both fractions, was 1 *μ*g/mL. For the fungus *Microsporus gypseum, Microsporus fulvum,*and *Microsporus gallinae, *none of the fraction presented inhibitory activity	[114]

Anti-inflammatory	Aerial parts	Methanol and petrol ether extracts	At 100 and 200 mg/kg/day, over 7 days, by oral route in mice, only the methanol extract presented significant anti-inflammatory activity on carrageenan-induced paw edema	[92]
	Leaves	Aqueous extract	At 100 and 200 *μ*g/mL, significantly prevented the lysis of human red blood cells in membrane stabilization method *in vitro *	[118]
		Ethanol extract	At 100 *μ*g/mL, significantly prevented the lysis of human red blood cells in membrane stabilization method *in vitro *	[118]
		Methanol extract and leaf paste	At 500 and 1000 mg/kg, by oral route in rats, inhibited the carrageenan-induced paw edema. At 50 and 100 mg/kg, by oral route in rats, inhibited the cotton pellet induced granuloma formation in rats. At 0.5 and 1 mg/ear, the leaf paste reduced the inflammation response in mouse ear edema model	[28]
	Not specified	Methanol and petrol ether extracts from bark	At 200 mg/kg, by oral route in rats, both extracts reduced the carrageenan-induced paw edema	[115]

Antimalarial	Leaves	Aqueous extract	30 *μ*g inhibited *in vitro* the growth of *Plasmodium falciparum *	[31]
		Dichloromethane extract	Active *in vitro* against *Plasmodium falciparum*, with IC_50_ of about 35 *μ*g/mL	[81]

Antineoplasic	Roots	Ethanolic extract and jatrophone	The ethanol extract, as well as jatrophone, exhibited significant inhibitory activity *in vitro* against cells derived from human carcinoma of the nasopharynx and lymphocytic leukemia P-388 and *in vivo* against four standard animal tumor systems	[66]
		Falodone	Showed potent proliferation inhibitory activity against A-549 human cancer cell line, with IC_50_ of 120 *μ*g/mL	[13]
		2*α*-Hydroxyjatrophone, 2*β*-hydroxy-5,6-isojatrophone and 2*β*-hydroxyjatrophone, diterpenes isolated from petrol ether extract	Presented antineoplasic activity upon P-388 lymphocytic leukemia test system both *in vivo* and *in vitro*, as well as for the Eagle's carcinoma of the nasopharynx test system *in vitro *	[64]
	Not specified	Abiodone	Not specified*	[73]

Antioxidant	Leaves	Methanol, ethyl acetate, and aqueous extract	All extracts showed significant antioxidant activity *in vitro* in DPPH free radical, ferric thiocyanate, and nitric oxide scavenging methods*	[55]
	Whole plant	Petrol ether, chloroform, ethyl acetate, and *n*-butanol extracts	All extracts showed only poor DPPH scavenging activity. The total antioxidant capacity was higher in ethyl acetate and *n*-butanol extracts, having the petrol ether and chloroform showing only poor activity. The lipid peroxidation was inhibited only partially by the extracts, with the ethyl acetate being the most active and the petrol ether being the least	[48]

Antispasmodic	Aerial parts	Ethanol extract, fractions, and subfractions	At 500, 1000, and 2000 mg/kg, by oral route in mice, showed significant antispasmodic activity in mouse intestinal transit model and at 0.5, 1.0, and 2.0 mg/mL inhibited *in vitro* the acetylcholine and calcium-induced contractions of isolated rat jejunum. Only the organic fraction of the extract had a calcium-antagonist effect, whereas both chloroformic and aqueous fractions had anticholinergic effect	[119]

Antiviral	Aerial parts	Water and ethyl acetate fractions from methanol extract	At 1, 10, and 100 *μ*g/mL, both fractions presented 100% of inhibition of plaque-forming ability of *Sindbis virus* in treatment preinfection protocol (IC_50_ < 1 *μ*g/mL), while in treatment postinfection, the IC_50_ of water fraction increased to 512 and acetate fraction increased to 37 *μ*g/mL. For murine cytomegalovirus, IC_50_ of 1.7 and 1.5 to water and ethyl acetate fractions were observed, respectively, in treatment preinfection protocol. In the treatment postinfection, however, no inhibition was observed in this microorganism	[114]
	Not specified	Methanol extract from barks	Partially active against *Sindbis virus* and herpes simplex virus-l. Inactive against human poliovirus	[107]

Bronchodilator	Stems	Aqueous extract	The extract was inactive in bronchodilator activity in guinea pigs	[120]

Contraceptive	Leaves	Ethanol extract	At 450 mg/kg/day, over 21 days, by oral route, caused an antifertility activity in female mice	[121]

Healing	Aerial parts	Ethanol 70% extract	At 1 mL/kg dose, by intraperitoneal route in rats, presented beneficial activity in healing process of colonic anastomosis	[122]
	Aerial parts	Ethanol 70% extract	At 200 mg/kg, by intraperitoneal route in rats, favored the healing process of gastrorrhaphies and reduced the acute inflammatory reaction *in vivo *	[123]
	Leaves	Hydroethanol extract	At 200 mg/kg, by intraperitoneal route, decreased the inflammation and increased vascular neoformation and collagen deposition when compared to the control group in healing process of sutures performed on the bladder of rats. However, in general, no favorable healing effect was observed.	[108]
	Not specified	Ethanolic extract	Although some improvement could be observed in suture healing of ventral abdominal wall of rats treated with 100 mg/kg of extract (intraperitoneal instillation intraperitoneal cavity), in general, only a poor healing activity was observed.	[124]
		Not specified	At 0.1 mL volume, by topical application, the crude extract presented significant differences concerning the macroscopic and microscopic aspects of healing process occurring in open skin lesions in rats	[125]

Hemostatic	Latex	Crude fresh latex	Decreased clotting and bleeding time in healthy subjects	[101]

Hepatoprotective	Aerial parts	Petrol ether, methanol, and water extracts	At 200 mg/kg/day, over 7 days, by oral route in rats, both extracts presented hepatoprotective activity in carbon tetrachloride induced liver damage, with the petrol ether being the most active and the methanol being the least	[126]

Hypotensive and vasorelaxant	Aerial parts	Ethanolic extract	At 125 and 250 mg/kg/day, over 4 weeks, by oral route in rats, in a dose-dependent manner, reduced the systolic blood pressure and produced a concentration-dependent relaxant effect in rat isolated (*ex vivo*) endothelium-deprived mesenteric artery precontracted with norepinephrine or CaCl_2_	[45]

Immunomodulatory	Whole plant	Petrol ether extract	At 100, 200, and 400 *μ*g/mL increased the proliferation of mouse spleen cell *in vitro *	[127]

Local anesthetic	Not specified	Methanol and aqueous extracts	Both extracts presented significant local anesthetic activity by plexus anesthesia in frogs*	[128]

Relaxant effect on uterine smooth muscle (tocolytic activity)	Aerial parts	Ethanolic extract and chloroformic and aqueous fractions	At 0.5 and 1.0 mg/mL, the ethanolic extract reduced the calcium-evoked contractile response of the uterine smooth muscle, as well as the chloroformic fraction. The aqueous fraction presented only slight effect	[129]

Sedative and anxiolytic	Fruits	Methanol extract	At 200 and 400 mg/kg, by oral route in mice, presented sedative effect in the hole cross test; At 200 mg/kg, presented anxiolytic activity in hole board test; At 400 mg/kg, presented anxiolytic activity in elevated plus-maze test	[110]
	Leaves	Methanol extract	At 200 and 400 mg/kg, by oral route in mice, presented sedative effect in the hole cross test; At 200 mg/kg, presented anxiolytic activity in hole board test; At 400 mg/kg, presented anxiolytic in elevated plus-maze test	[111]

*The complete version of the paper was not accessible, so the information was obtained from its abstract.

IC_50_: concentration that inhibits 50% of the referred activity.
